# *Coxiella burnetii* Affects HIF1α Accumulation and HIF1α Target Gene Expression

**DOI:** 10.3389/fcimb.2022.867689

**Published:** 2022-06-09

**Authors:** Inaya Hayek, Manuela Szperlinski, Anja Lührmann

**Affiliations:** Mikrobiologisches Institut – Klinische Mikrobiologie, Immunologie and Hygiene, Universitätsklinikum Erlangen, Friedrich-Alexander-Universität (FAU) Erlangen-Nürnberg, Erlangen, Germany

**Keywords:** *Coxiella burnetii*, HIF1α, T4SS, metabolism, apoptosis, inflammation

## Abstract

HIF1α is an important transcription factor regulating not only cellular responses to hypoxia, but also anti-infective defense responses. We recently showed that HIF1α hampers replication of the obligate intracellular pathogen *Coxiella burnetii* which causes the zoonotic disease Q fever. Prior to development of chronic Q fever, it is assumed that the bacteria enter a persistent state. As HIF1α and/or hypoxia might be involved in the induction of *C. burnetii* persistence, we analyzed the role of HIF1α and hypoxia in the interaction of macrophages with *C. burnetii* to understand how the bacteria manipulate HIF1α stability and activity. We demonstrate that a *C. burnetii*-infection initially induces HIF1α stabilization, which decreases then over the course of an infection. This reduction depends on bacterial viability and a functional type IV secretion system (T4SS). While neither the responsible T4SS effector protein(s) nor the molecular mechanism leading to this partial HIF1α destabilization have been identified, our results demonstrate that *C. burnetii* influences the expression of HIF1α target genes in multiple ways. Therefore, a *C. burnetii* infection promotes HIF1α-mediated upregulation of several metabolic target genes; affects apoptosis-regulators towards a more pro-apoptotic signature; and under hypoxic conditions, shifts the ratio of the inflammatory genes analyzed towards a pro-inflammatory profile. Taken together, *C. burnetii* modulates HIF1α in a still elusive manner and alters the expression of multiple HIF1α target genes.

## Introduction

Hypoxia-inducible factor (HIF)-1 was first recognized as an essential regulator of cellular responses to limited oxygen availability (Majmundar et al., 2010). Recent research has shown that HIF1 activity is also critical for shifting cellular metabolism, regulating immune cell activity, and mounting anti-infective defense responses (Cramer et al., 2003; Knight and Stanley, 2019). HIF1 is a heterodimer, consisting of HIF1α and HIF1β (Wang et al., 1995). The activity of the complex is controlled by proteasomal degradation of the α-subunit. Thus, prolyl hydroxylases (PHDs) hydroxylate HIF1α, which mediates binding to the von Hippel-Lindau (VHL) E3 ubiquitin ligase and leads to proteasomal degradation of HIF1α (Maxwell et al., 1999; Ohh et al., 2000; Jaakkola et al., 2001). Importantly, PHDs require oxygen, Fe^2+^ and 2-oxoglutarate for HIF1α hydroxylation [reviewed in: (Greer et al., 2012; Hayek et al., 2021)]. Therefore, in the absence of oxygen, its co-factors or co-substrates, HIF1α is stabilized. However, HIF1α stabilization can also occur under normoxic conditions (in the presence of oxygen) in response to increased levels of the TCA cycle intermediates succinate or fumarate, or in the presence of nitric oxide (NO) (Hewitson et al., 2007; Tannahill et al., 2013; Mills et al., 2016). In addition, bacterial, viral, fungal, and parasitic infections might also induce HIF1α stabilization (Devraj et al., 2017; Knight and Stanley, 2019). Once the heterodimer is formed, it attaches to the promoter region of genes containing the hypoxia response element (HRE) and induces their transcription. In addition, HIF1 interacts with other signaling pathways (including Notch, Wnt and Myc) in an HRE-independent manner (Koshiji et al., 2004; Gustafsson et al., 2005; Kaidi et al., 2007; Semenza, 2014; Strowitzki et al., 2019). Thereby, HIF1 regulates transcription of genes involved in metabolic reprogramming, immune responses, and anti-infectious activity (Obach et al., 2004; Kelly and O'Neill, 2015; Devraj et al., 2017).

Hypoxia, a state of insufficient oxygen availability, impairs several important antimicrobial defense mechanisms. To control bacterial infections under hypoxia, myeloid cells induce the production of anti-microbial peptides and pro-inflammatory cytokines, deplete essential metabolites, and modulate their phagocytic capacity and phagosome maturation (Hayek et al., 2021). Under these conditions, some bacteria are controlled under hypoxia and/or HIF1α, while other pathogens survive or even replicate.

We recently showed that in hypoxic murine macrophages, HIF1α or HIF1α -mediated signaling impedes *C. burnetii* replication (Hayek et al., 2019). This obligate intracellular bacterium is a zoonotic pathogen. Its primary reservoir are domestic ruminants such as cattle, sheep and goats ( Maurin and Raoult, 1999). Although infected ruminants are mainly asymptomatic, in pregnant animals the infection might lead to abortion, premature delivery or stillbirth. Infected animals shed the pathogen through birthing products, feces or milk which are the main source for human infection (Van den Brom et al., 2015). Although often asymptomatic, Q fever may manifest in humans as an acute disease (mainly as a self-limited febrile illness, pneumonia, or hepatitis) or as a chronic disease (mainly endocarditis). Importantly, chronic Q fever develops several months or years after the primary infection (Anderson et al., 2013). A short-term treatment with doxycycline is still considered the mainstay of antibiotic therapy of acute Q fever, whereas chronic Q fever patients have to be treated with doxycycline in combination with hydroxyl chloroquine for at least 18 months. Thus, a more efficient therapy to treat chronic Q fever has to be developed. In addition, it is crucial to increase our knowledge of chronic Q fever development, especially since it develops months or years after the primary infection, during which the patient does not show any symptoms, suggesting a prolonged state of bacterial persistence (Harris et al., 2000; Sukocheva et al., 2016). Our previous results suggest that in macrophages, HIF1α is required for impeding *C. burnetii* replication by impairing STAT3 activation, which results in reduced levels of the TCA intermediate citrate (Hayek et al., 2019). Importantly, bacterial viability was maintained allowing bacterial persistence. Thus, HIF1α might play an important role in the induction of *C. burnetii* persistence, and consequently, the development of chronic Q fever. Therefore, we aim to analyze the roles of HIF1α and hypoxia for the interaction of macrophages with *C. burnetii* in more detail.

## Materials and Methods

### Reagents and Cell Lines

Bone marrow derived macrophages from C57BL/6 “J” male mice (Charles River; Strain Code: 027) were prepared as described (Hayek et al., 2019). Briefly, bone marrow cells were extracted from femur and tibia of at least 6 weeks old mice and propagated in sterile Teflon bags (Angst+Pfister) containing DMEM + GlutaMax (Thermo Fisher), 10% Fetal Calf Serum (FCS) (Biochrom), 5% Horse Serum (Cell Concepts), 1% MEM Non-Essential Amino Acids Solution (Life Technologies), 0.5% HEPES (AppliChem) and 20% supernatant of L929 cells for 7-10 days at 37°C, 10% CO_2_ and 21% O_2_. Macrophages were cultured for infection experiments in CMoAB medium, consisting of RPMI 1640 medium (Thermo Fisher) supplemented with 10% FCS, 1% HEPES and 0.5% β-mercaptoethanol (Sigma Aldrich). Murine macrophages were seeded and left to adhere for 1 to 2 h at 37°C, 5% CO_2_, 21% O_2_ (normoxia) prior to infection.

### *C. burnetii* Cultivation

All *C. burnetii* strains used in this study were inoculated at a concentration of 1 x 10^6^ C*. burnetii*/ml in ACCM-2 (Sunrise Science Products, Cat#4700-300) medium and cultivated for 5 days at 37°C, 5% CO_2_, and 2.5% O_2_. The *C. burnetii* Nine Mile phase II (NMII) clone 4 (RSA439) served as wild type (wt) strain in this study. When growing *C. burnetii* Δ*dotA* (Schäfer et al., 2020) or the Δ*dotA C. burnetii* transposon mutant (kindly provided by Matteo Bonazzi (Martinez et al., 2014)), 3 μg/ml chloramphenicol was added to the axenic medium. *C. burnetii* NMII was heat-killed (Hk wt) at 70°C for 30 min under shaking at 500 rpm.

### *E. coli* Cultivation

*E. coli* DH5α were plated on a Luria broth (LB) agar plate and placed overnight at 37°C. A single colony was picked to inoculate 3 ml LB medium, which was left to rotate for 5 h at 37°C. Then, 50 µl of the liquid culture were transferred into 3 ml of fresh LB medium and rotated overnight at 37°C.

### Infection

To adjust *C. burnetii* infection concentrations, the optical density at OD_600_ was measured, with an OD_600_ of 1 equaling 1 x 10^9^ C*. burnetii*/ml. To adjust *E. coli* infection concentrations, the optical density at OD_600_ was measured, where an OD_600_ of 1 equals 8 x 10^8^ *E. coli*/ml. Unless otherwise mentioned, macrophages were infected with *C. burnetii* or *E. coli* at an MOI (multiplicity of infection) of 10. After macrophage seeding, the cells were infected with the bacteria and placed under normoxia or 0.5% O_2_ (hypoxia) for 4 h at 37°C, 5% CO_2_. At the 4h time point, the cells were either harvested or the medium was discarded and replaced with fresh CMoAB for the later time points.

### Treatment With LPS

The concentration of *E. coli* LPS (Sigma, L4391) was adjusted in CMoAB at 100 ng/ml. After macrophage seeding, the cells were treated with LPS and placed under normoxia or hypoxia. After 4 h, the medium was discarded and replaced with fresh CMoAB. Samples were harvested 24 h post-infection.

### Treatment With Chemicals

Chloramphenicol (Roth) was adjusted to a concentration of 25 μg/ml in CMoAB and then applied to the infected macrophages to induce bacterial growth arrest along the course of infection.

### Hypoxia

Hypoxic conditions were set to 0.5% O_2_ and 5% CO_2_ at 37°C in an InvivO2 hypoxic chamber (Baker Ruskinn). Media and buffers were equilibrated at least 4 h in the hypoxic chamber before starting an experiment.

### Harvesting Protein Samples for Immunoblots

For HIF1α and actin immunoblot samples, uninfected, infected or LPS-treated macrophages were lysed with 10 mM Tris-HCl pH 6.8, 6.65 M Urea, 10% Glycerol, 1% SDS with freshly added 1 mM DTT and cOmplete Mini Protease Inhibitor Cocktail (Roche, Cat#04693124001). Hypoxic samples were harvested in the hypoxic chamber to prevent HIF1α destabilization. The samples were mixed for 30 s with the homogenizer unit (VWR) and corresponding pestles (VWR). Finally, 20 μL 4x Laemmli SDS buffer was added to the samples, which were then heated at 85°C for 8 min, shaking at 450 rpm.

### Immunoblot

Proteins were separated by SDS-PAGE using 4-12% Bis-Tris Gels (Thermo Fischer Scientific) and transferred to a PVDF membrane (Merck Millipore). The membranes were probed with primary antibodies directed against HIF1α (Cayman 10006421/Biomol) or actin (Sigma-Aldrich A2066). The proteins were visualized by using the secondary antibody α - Rabbit IgG (H+L)-HRP (Jackson ImmunoResearch Labs, Cat#111-035-045) and a chemiluminescence detection system (Thermo Fisher). Densitometric analysis was performed using ImageJ (NIH).

### Immunofluorescence

The experimental steps of immunofluorescence staining were described in detail elsewhere (Hayek et al., 2019). Briefly, macrophages were seeded on 10 mm sterile coverslips in 24-well plates. After infection and incubation, the cells were fixed with 4% paraformaldehyde (PFA) and permeabilized with ice-cold methanol. The cells were then quenched with 50 mM NH_4_Cl in PBS/5% goat serum (GS) followed by incubation with the primary antibody against *C. burnetii* NMII (Davids Biotechnology). Alexa Fluor 594 (Jackson ImmunoResearch Labs) was used as the secondary antibody. Finally, the slides were mounted with ProLong Diamond containing DAPI (Invitrogen). Immunofluorescent images were taken using the Carl Zeiss LSM 700 Laser Scan Confocal Microscope and the ZEN2009 software.

### RNA

RNA samples were harvested with peqGOLD TriFast (Peqlab VWR, Cat#30-2010) or the RNeasy Plus Kit (Qiagen) and isolated according to manufacturer’s protocol. Isolated RNA was treated with DNase and RDD buffer (QIAGEN, Cat#79254) for 10 min at 37°C, followed by DNase inactivation at 75°C for 5 min. The successful removal of any DNA contamination was confirmed by PCR analysis. Next, first strand cDNA was synthesized from the DNase-treated RNA with SuperScript II Reverse Transcriptase (Invitrogen by Life Technologies, Cat#18064-022) according to manufacturer’s protocol. The resulting cDNA was diluted 5-fold (final concentration of about 100 ng) and served as template in qPCR using the QuantiFast SYBR Green PCR Kit (QIAGEN, Cat#204054), along with a final concentration of 100 nM of each primer in a final volume of 10 µL per reaction. Murine hypoxanthine guanine phosphoribosyl transferase (*mHprt1*) was the housekeeping gene. The sequence of the primer pairs used to investigate gene expression (*HIF1α*, *PHD1*, *PHD2*, *PHD3*, *VHL*, *IL1β*, *Nos2*, *IL10*, *IL6*, *PKM2*, *LDHA*, *Glut1*, *PDK1*, *Bcl2*, *Bax*, *Trp53*, *Becn1*, *Bnip3*, *Bnip3l*, *P300*, *FIH*, and *CBP*) are listed in **Table 1**. The expression levels of these genes were quantified by referencing to *mHPRT1* and normalizing to the uninfected or wt-infected normoxic sample. To calculate the fold change, the 2^-(ΔΔCT) method was applied.

**Table 1:** Primers used.

Name	Direction	5’ to 3’ sequence
HPRT1	forward	TCCTCCTCAGACCGCTTTT
HPRT1	reverse	CCTGGTTCATCATCGCTAATC
HIF1α	forward	CATCATCTCTCTGGATTTTGGCAGCG
HIF1α	reverse	GATGAAGGTAAAGGAGACATTGCCAGG
PHD2	forward	GCGGGAAGCTGGGCAACTAC
PHD2	reverse	CCATTTGGGTTATCAACGTGACGGAC
PHD3	forward	GGCCGCTGTATCACCTGTATCTACTAC
PHD3	reverse	CAGAAGTCTGTCAAAAATGGGCTCCAC
PHD1	forward	GTAATCCGCCACTGTGCAGGG
PHD1	reverse	CATCGCCGTGGGGATTGTCAAC
VHL	forward	GCCATCCCTCAATGTCGATGGAC
VHL	reverse	GACGATGTCCAGTCTCCTGTAGTTCTC
IL1β	forward	GTGCTGTCGGACCCATATGAGC
IL1β	reverse	CCCAAGGCCACAGGTATTTTGTCG
Nos2	forward	GACCAGAGGACCCAGAGACAAGC
Nos2	reverse	GCTTCCAGCCTGGCCAGATG
IL10	forward	TCAGCAGGGGCCAGTACAGC
IL10	reverse	GCAGTATGTTGTCCAGCTGGTCC
IL6	forward	AGACTTCCATCCAGTTGCCTTCTTGG
IL6	reverse	GTCTGTTGGGAGTGGTATCCTCTGTG
PKM2	forward	GACCTGAGATCCGGACTGGACTC
PKM2	reverse	GCAGATGTTCTTGTAGTCCAGCCAC
LDHA	forward	GGATCTCCAGCATGGCAGCC
LDHA	reverse	CTCTCCCCCTCTTGCTGACGG
Glut1	forward	GCTGTGGGAGGAGCAGTGC
Glut1	reverse	TGGATGGGATGGGCTCTCCG
PDK1	forward	CCTTAGAGGGCTACGGGACAGATG
PDK1	reverse	CACCAGTCGTCAGCCTCGTG
Bcl2	forward	TGACTGAGTACCTGAACCGGCATC
Bcl2	reverse	CCAGGCTGAGCAGGGTCTTCA
Bcl2	forward	GACAACATCGCCCTGTGGATGAC
Bcl2	reverse	TCAAACAGAGGTCGCATGCTGG
Bax	forward	GCCCCAGGATGCGTCCAC
Bax	reverse	GAGTCCGTGTCCACGTCAGC
Trp53	forward	CTGGGCTTCCTGCAGTCTGG
Trp53	reverse	ACCCACAACTGCACAGGGC
Becn1	forward	CTCGCCAGGATGGTGTCTCTCG
Becn1	reverse	GAGTCTCCGGCTGAGGTTCTCC
Bnip3	forward	GCCCAGCATGAATCTGGACGAAG
Bnip3	reverse	CTCGCCAAAGCTGTGGCTGTC
Bnip3l	forward	GCAGACTGGGTATCAGACTGGTCC
Bnip3l	reverse	GGCTCCACTCTTCCTCATGCTTAGAG
P300	forward	GCTTGCGGACTGCAGTCTATCATG
P300	reverse	CTGGGTGGACAGGCCCAGA
FIH	forward	GGGCAGCTGACCTCTAACCTGTT
FIH	reverse	AGGCACTCGAACTGATCCGGAG
CBP	forward	CACATGACACATTGTCAGGCTGGG
CBP	reverse	CAGGACAGTCATGTCGTGTGCAG

### Primers

The primers used are listed in **Table 1**.

### Statistical Analysis

Using GraphPad Prism 5, statistical analysis of the presented data was performed. As mentioned in the individual figure legends, a one sample t-test or an unpaired two-tailed Student’s t test was used. The one-sample t-test was used when comparing datasets to normalized values. A value of p < 0.05 was considered significant.

## Results

### *C. burnetii* Infection Augments Hypoxia-Induced HIF1α Stabilization

**Figure 1:**
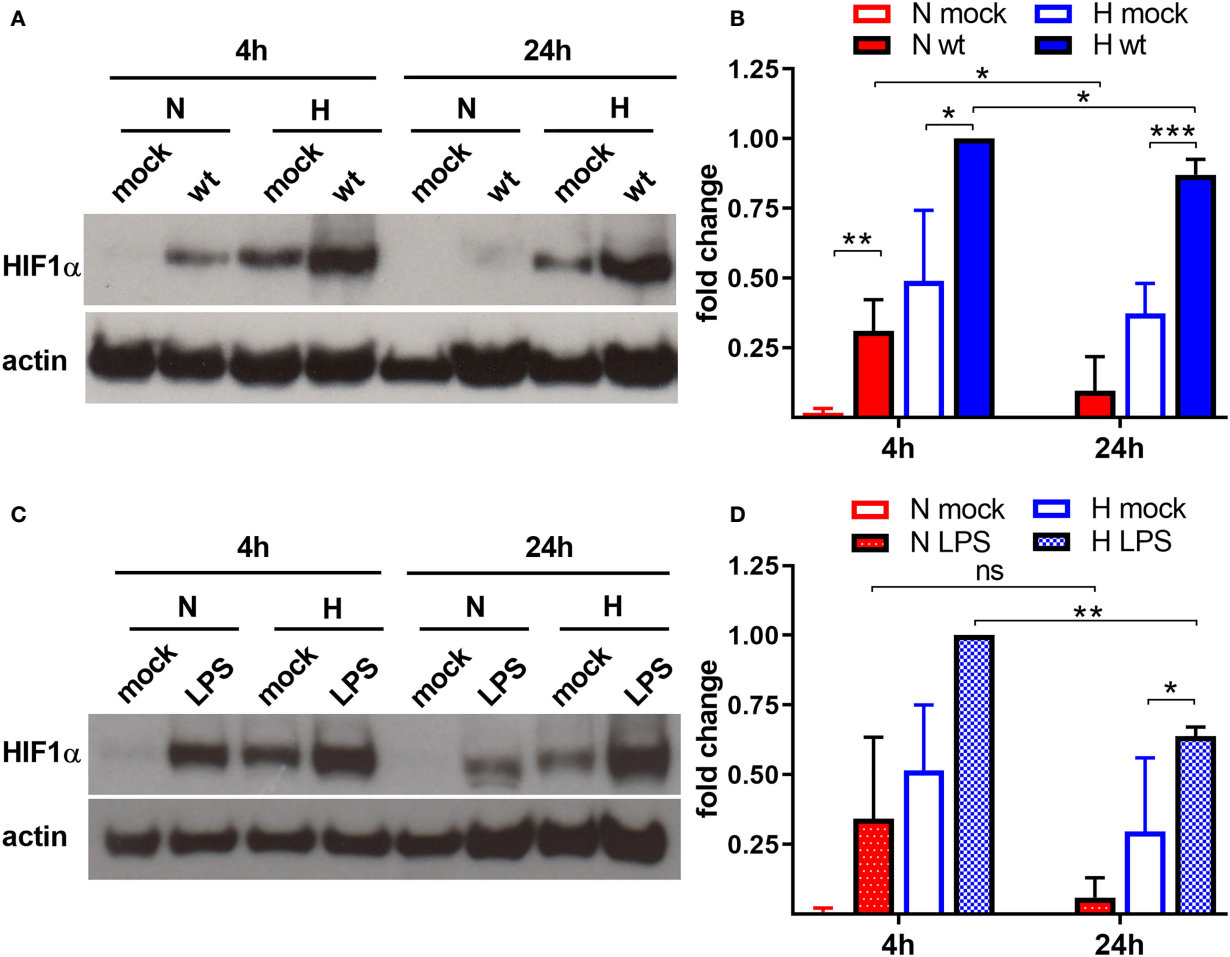
*C. burnetii* and LPS boost HIF1α stabilization. **(A, B)** Murine BMDM either uninfected (mock) or infected with *C. burnetii* (wt) for 4 and 24 h under normoxia (N) or hypoxia (H) were analyzed by immunoblot analysis using antibodies against HIF1α and actin as loading control. **(A)** One representative experiment out of four independent experiments is shown. **(B)** Densitometric analysis of the HIF1α/actin ratio was performed using ImageJ. Fold changes are shown relative to cells infected for 4 hours under (H) Mean ± SD, n = 4, one-sample t test or t test. ***p < 0.001, **p < 0.01, *p < 0.05. **(C, D)** Murine BMDM either untreated (mock) or treated with LPS (100 ng/ml) for 4 and 24 h under N or H were analyzed by immunoblot analysis using antibodies against HIF1α and actin as loading control. **(C)** One representative experiment out of three independent experiments is shown. **(D)** Densitometric analysis of the HIF1α/actin ratio was performed using ImageJ. Fold changes are shown relative to cells treated with LPS for 4 hours under (H) Mean ± SD, n = 3, one-sample t test or t test. **p < 0.01, *p < 0.05, ns = non-significant.

**Figure 2:**
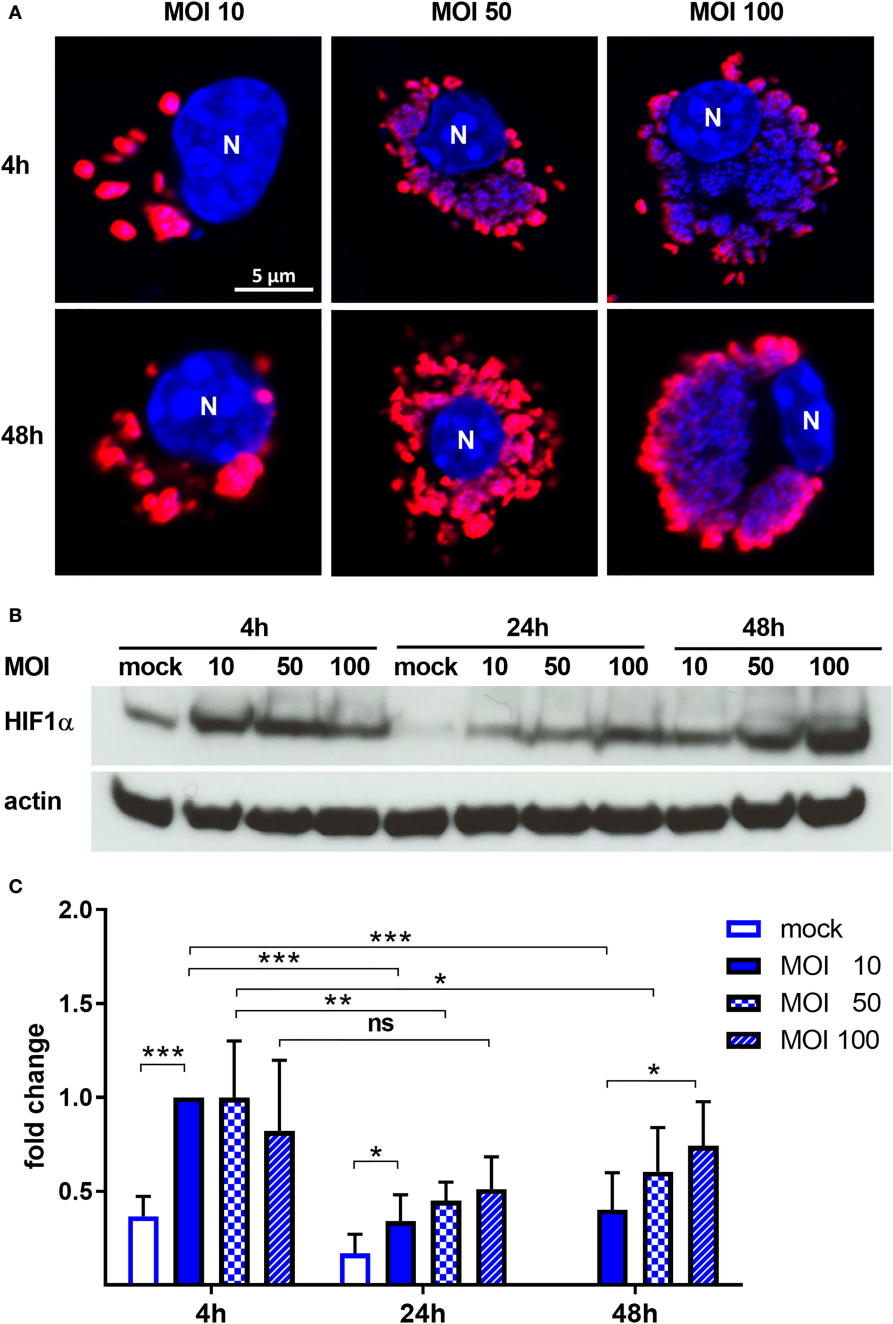
*C. burnetii* intensifies HIF1α stabilization under hypoxia in a dose-dependent manner. **(A)** Representative immunofluorescence micrographs of murine BMDM infected with *C. burnetii* for 4 and 48 h under hypoxia at MOI 10, 50 or 100. The cells were fixed, permeabilized and stained with DAPI (blue) and anti-*C. burnetii* (red). N = nucleus. **(B, C)** BMDM infected with *C. burnetii* for 4, 24 and 48 h under hypoxia at MOI 10, 50 or 100 were analyzed by immunoblot using antibodies against HIF1α and actin as loading control. Importantly, uninfected BMDMs were only cultivated for 24 h under hypoxia, as cell viability was significantly reduced at later time points. **(B)** One representative immunoblot from 4 independent experiments is shown. **(C)** Densitometric analysis of the HIF1α/actin ratio was performed using ImageJ. Fold changes are shown relative to cells infected with MOI of 10 for 4 hours. Mean ± SD, n=6, one-sample t-test or t-test. ***p < 0.001, **p < 0.01, *p < 0.05, ns = p > 0.05.

Previous experiments suggested that *C. burnetii* increases the HIF1α protein level under hypoxic conditions (Hayek et al., 2019). As HIF1α accumulation is responsible for inhibiting *C. burnetii* replication, we aimed to determine whether *C. burnetii* is capable of modulating the HIF1α protein level. Thus, we infected bone marrow derived macrophages (BMDM) with *C. burnetii* at an MOI of 10 for 4 or 24 hours under normoxic (21% O_2_, 5% CO_2_) or under reduced oxygen (0.5% O_2_, 5% CO_2_) conditions. In the following, we refer to this reduced oxygen condition as hypoxia. We analyzed the HIF1α protein level of the infected cells, kept under different oxygen conditions, by immunoblot analysis. Under normoxia, HIF1α is constantly degraded. However, the infection with *C. burnetii* for 4 h resulted in transient stabilization of HIF1α, which was almost absent at 24 h post-infection (**Figures 1A, B**). Under hypoxia, we observed HIF1α protein accumulation in uninfected cells at 4 and 24 h, which was further augmented by infection with *C. burnetii*. Importantly, lipopolysaccharide (LPS) stimulation also led to HIF1α stabilization under normoxia and, more pronounced, under hypoxia (**Figures 1C, D**). Thus, the *C. burnetii*-mediated HIF1α stabilization might be partially due to the recognition of LPS. However, we used LPS from *E. coli*. Thus, the comparison has to be taken with caution, as the lipid A of *C. burnetii* LPS differs significantly from enterobacterial lipid A and fails to signal via toll-like receptor (TLR) 2 and 4 (Zamboni et al., 2004; Abnave et al., 2017; Beare et al., 2018). The fact, that *C. burnetii* infection increased HIF1α stabilization, prompted us to analyze the impact of bacterial load on HIF1α stabilization. Therefore, we infected BMDM at an MOI of 10, 50 or 100 under hypoxia and analyzed the infection by immunofluorescence and the HIF1α protein level by immunoblot. As shown in **Figure 2A**, the increased MOI led to a higher bacterial load. Importantly, the increase in bacterial load did not result in increased HIF1α stabilization at 4 h post-infection (**Figure 2B**). However, at 24 h and 48 h post-infection, the increasing infection dose seemed to result in higher HIF1α protein levels (**Figures 2B, C**). Moreover, at an MOI of 10, HIF1α stabilization decreases during the course of the infection. Thus, not only the oxygen level influences HIF1α stabilization, but also the pathogen seems to modulate this important transcription factor. This hypothesis prompted us to determine whether bacterial viability is required for affecting HIF1α stabilization.

### *C. burnetii* Curtails Infection-Induced HIF1α Stabilization

**Figure 3:**
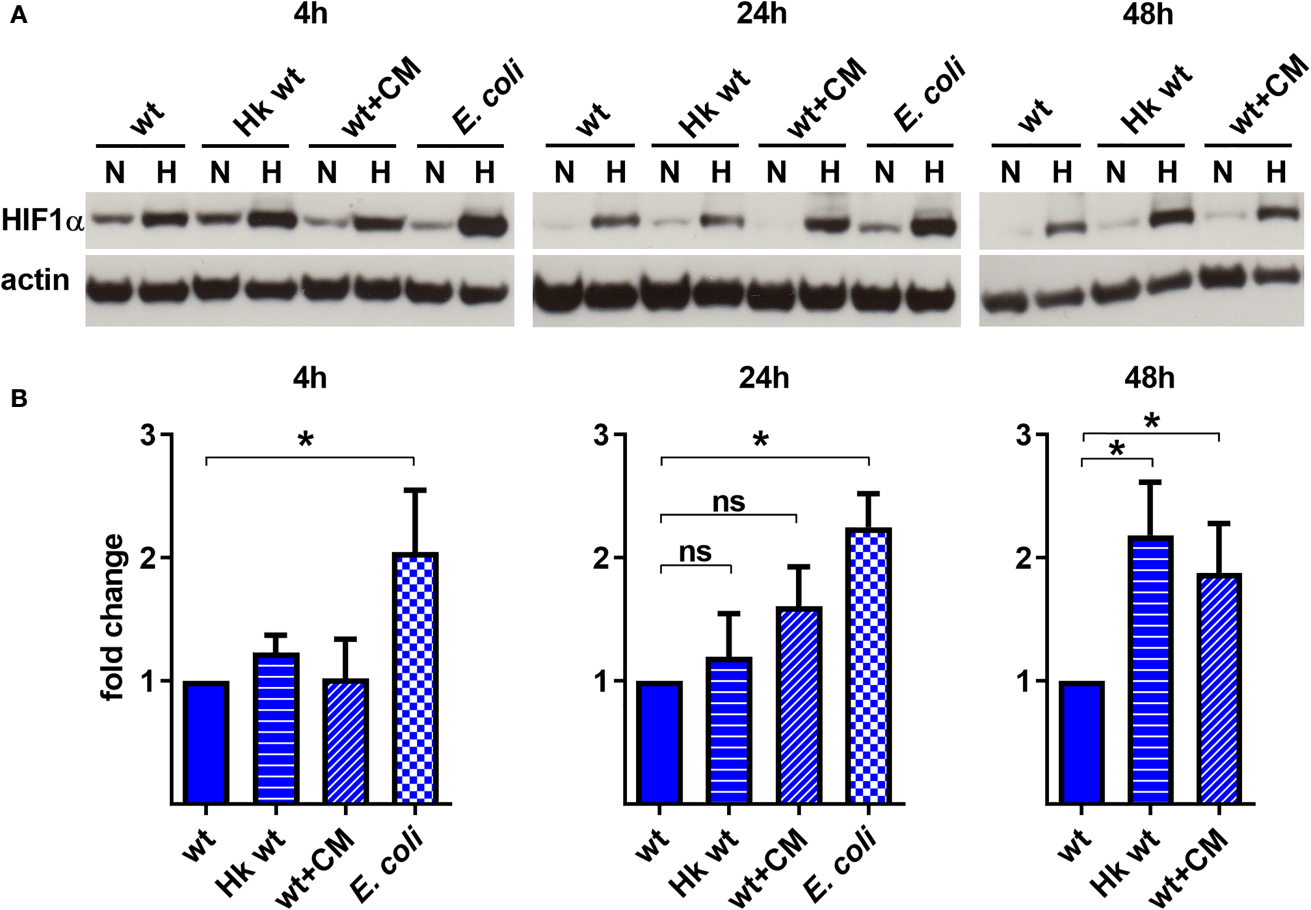
*C. burnetii*-mediated reduction of infection-induced HIF1α depends on bacterial viability. **(A, B)** Murine BMDM infected with *C. burnetii* (wt), heat-killed *C. burnetii* (Hk), chloramphenicol-treated *C. burnetii* (CM, 25 µg/ml) or *E. coli* for 4, 24 and 48 h under normoxia (N) or hypoxia (H) were analyzed by immunoblot using antibodies against HIF1α and actin as loading control. Importantly, *E. coli* infected BMDMs were only cultivated for 24 h under hypoxia, as cell viability was significantly reduced at later time points. **(A)** One representative immunoblot from 4 independent experiments is shown. **(B)** Densitometric analysis of the HIF1α/actin ratio was performed using ImageJ. Fold changes under hypoxia (H) are shown relative to cells infected with viable bacteria. Mean ± SD, n=4, one-sample t-test. *p < 0.05, ns=p > 0.05.

To analyze the role of *C. burnetii* in HIF1α stabilization, we infected BMDM at an MOI of 10 with either untreated *C. burnetii*, heat-killed *C. burnetii*, *C. burnetii* treated with chloramphenicol to inhibit bacterial protein synthesis or *E. coli* at an MOI of 10. In normoxic BMDM, *C. burnetii* led to HIF1α stabilization only at 4 h post-infection (**Figure 3A**). As this was observed regardless of the viability or physiological state of the pathogen, we assumed that this might be the reaction of the host cell to a pathogen associated molecular pattern (PAMP). This is in line with observations that microbial products, such as LPS, induce HIF1α accumulation also in the presence of O_2_ (Blouin et al., 2004; Werth et al., 2010). The fact that HIF1α is degraded in infected normoxic macrophages at later time points of infection might be due to the intracellular lifestyle of *C. burnetii*, which hides in an intracellular vacuole (Pechstein et al., 2017). Importantly, under hypoxic conditions the infection with *E. coli* induced a higher HIF1α protein level at 4 and 24 h post-infection compared to the infection with viable *C. burnetii*, indicating that *C. burnetii* might be able to restrict HIF1α accumulation. Similarly, at 48 h post-infection, the HIF1α protein level was increased in hypoxic BMDM infected with heat-killed *C. burnetii* compared to BMDM infected with viable *C. burnetii* (**Figures 3A, B**). We hypothesized that *C. burnetii* might be able to actively curtail HIF1α accumulation under hypoxic conditions. The observation that hypoxic BMDM infected with chloramphenicol-treated bacteria showed an increased HIF1α level too, suggests that bacterial protein synthesis is important for *C. burnetii*-mediated restriction of HIF1α.

### *C. burnetii* Reduces HIF1α Accumulation in a T4SS-Dependent Manner

**Figure 4:**
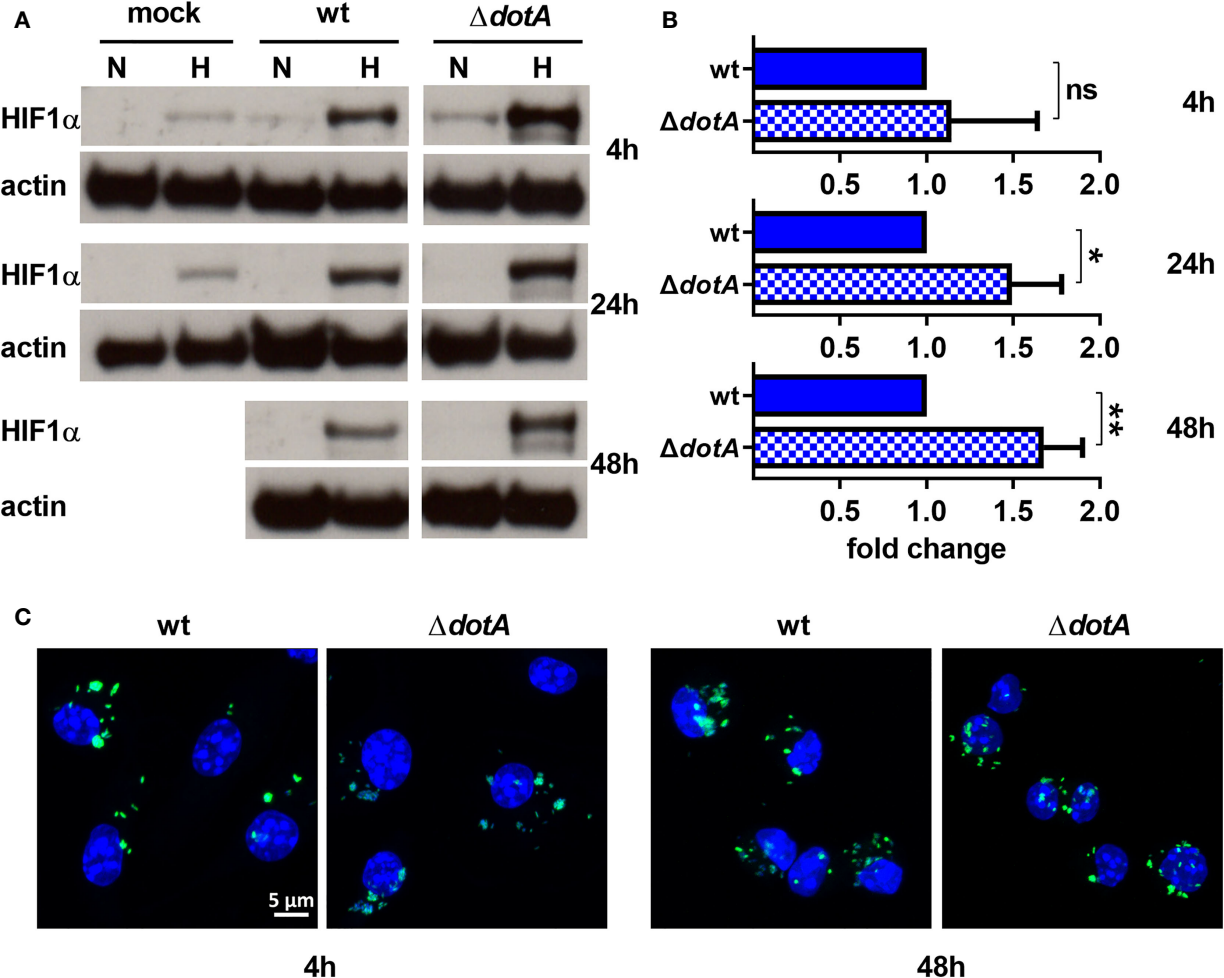
*C. burnetii* reduces HIF1α levels in a T4SS-dependent manner. **(A, B)** Murine BMDM either uninfected (mock) or infected with *C. burnetii* (wt) or *C. burnetii* lacking a functional T4SS (Δ*dotA*) for 4, 24 and 48 h under normoxia (N) or hypoxia (H) were analyzed by immunoblot using antibodies against HIF1α and actin as loading control. Importantly, uninfected BMDMs were only cultivated for 24 h under hypoxia, as cell viability was significantly reduced at later time points. **(A)** One representative Western blot from 4 independent experiments is shown. **(B)** Densitometric analysis of the HIF1α/actin ratio was performed using ImageJ. Fold changes under hypoxia (H) are shown relative to cells infected with wt. Mean ± SD, n=4, one-sample t-test. **p < 0.01, *p < 0.05, ns=p > 0.05. **(C)** Representative immunofluorescence micrographs of murine BMDM infected with *C. burnetii* (wt) (green) or the T4SS transposon mutant (Δ*dotA*) (green) for 4 and 48 h under hypoxia are shown. The cells were fixed, permeabilized and stained with DAPI (blue).

*C. burnetii* utilizes a type IV secretion system (T4SS) to inject bacterial effector proteins into the host cell to modify host cell pathways for the benefit of the pathogen (Lührmann et al., 2017). Bacteria lacking a functional T4SS are unable to replicate intracellularly, confirming that T4SS-driven modulation of host cell pathways is essential (Beare et al., 2011; Carey et al., 2011). Importantly, inhibition of bacterial protein synthesis by chloramphenicol-treatment also impairs T4SS function (Pan et al., 2008). Therefore, we asked whether the ability of *C. burnetii* to reduce HIF1α protein level under hypoxic conditions depends on the T4SS. We focused on hypoxic conditions, as, under normoxia, HIF1α is degraded starting at 24 h post-infection regardless of the pathogen viability (**Figure 3A**). Four hours of infection with the wild-type and the T4SS mutant (Δ*dotA*) similarly augmented hypoxia-induced HIF1α stabilization. Starting from 24 h post-infection, we detected increased HIF1α stabilization in hypoxic BMDM infected with the Δ*dotA* mutant (**Figures 4A, B**). Importantly, this was not mediated by differences in replication ability, as we did not observe any bacterial replication during the course of infection (**Figure 4C**). This is in line with our previous results, showing that *C. burnetii* is unable to replicate in hypoxic BMDM (Hayek et al., 2019). Taken together, our results suggest that *C. burnetii* infection results in HIF1α stabilization under hypoxia. However, viable *C. burnetii* are able to control HIF1α level in a T4SS-dependent manner.

### The T4SS Is Dispensable for *C. burnetii*-Induced Transcriptional Modulation of *HIF1α* and *PHD1*

**Figure 5:**
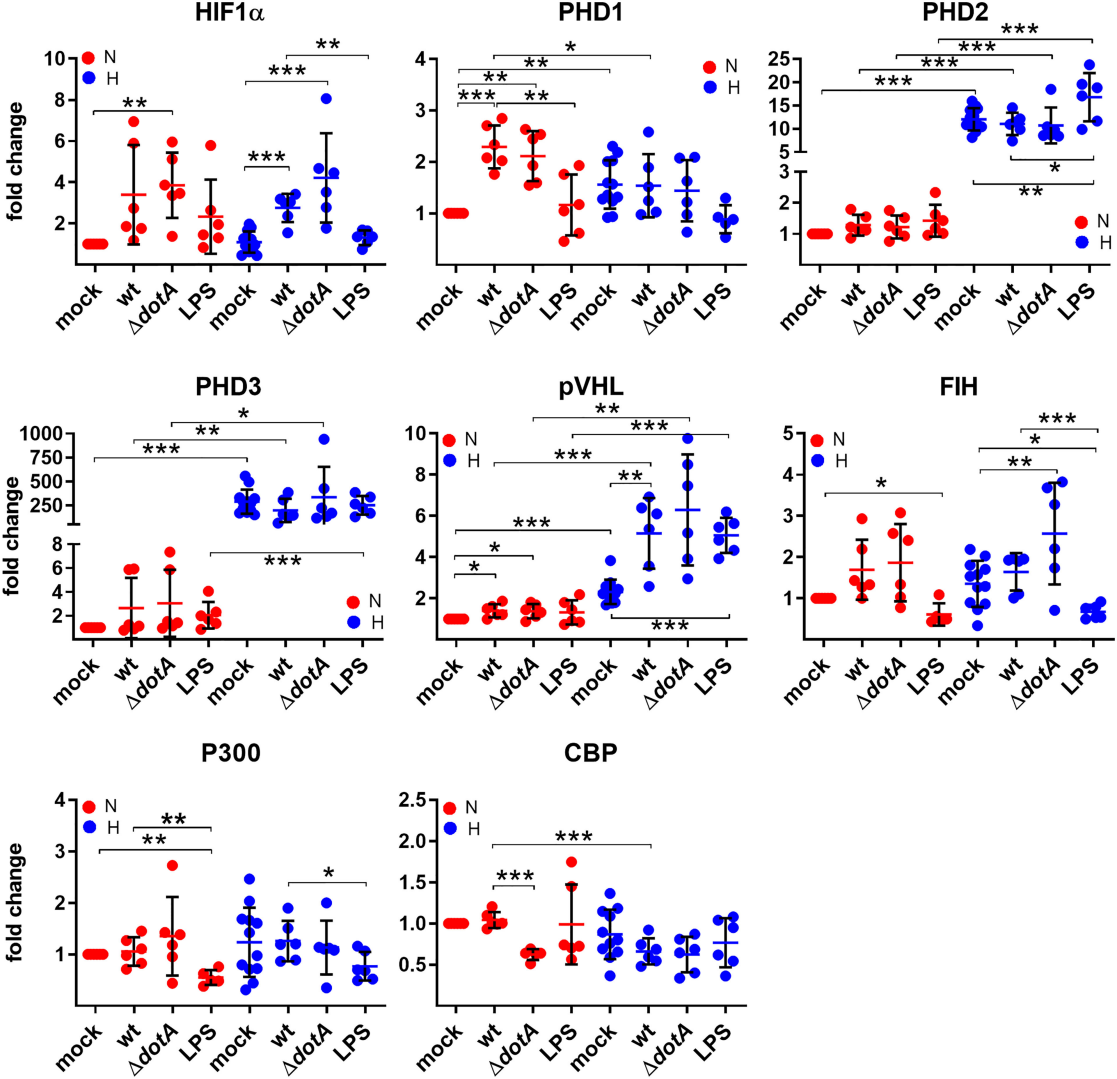
*C. burnetii* only marginally influences the expression of genes regulating HIF1α stability. BMDM were either uninfected (mock), infected with *C. burnetii* (wt), the T4SS transposon mutant (Δ*dotA*), or treated with LPS (100 ng/ml) for 24 h under normoxia (N) and hypoxia (H). Using qRT-PCR, the gene expression of murine *HIF1α*, *PHD1*, *PHD2*, *PHD3*, *VHL*, *FIH*, *p300* and *CBP* was analyzed. The data are displayed as Mean ± SD of 2^-ΔΔCT values (using murine *HPRT1* as a calibrator). Fold changes are shown relative to uninfected cells under N. The data shown for each of the *C. burnetii* (wt and Δ*dotA*) infection experiment and the LPS treatment experiment represent 3 independent experiments with biological duplicates. One sample t test or t test, n=5-6. ***p < 0.001, **p < 0.01, *p < 0.05.

To determine how *C. burnetii* might be able to manipulate HIF1α stabilization, we first analyzed the mRNA levels of HIF1α, factors influencing HIF1α degradation and factors important for HIF1α transcription activation by qRT-PCR. The degradation of HIF1α is controlled by prolyl hydroxylases (PHDs), which hydroxylate HIF1α, leading to the recruitment of the von Hippel-Lindau (VHL) E3 ubiquitin ligase, that ubiquitinates HIF1α targeting it for proteasomal degradation (Maxwell et al., 1999; Ohh et al., 2000; Jaakkola et al., 2001). Other factors influence HIF1α transcriptional activity: Factor Inhibiting HIF (FIH) hydroxylates HIF, preventing recruitment of p300 and CREB-binding protein (CBP) (Mahon et al., 2001), which are important for maximal transcriptional activation of HIF (Arany et al., 1996; Dyson and Wright, 2016; Pugh, 2016). A *C. burnetii* infection, but not LPS stimulation, led to upregulation of *HIF1α* expression under hypoxia regardless of the pathogen´s genotype (wild-type or Δ*dotA*) (**Figure 5**). The expression of the *PHDs* was affected differently by hypoxia. While the *PHD1* expression level was slightly increased by hypoxia, the levels of *PHD2* and *PHD3* were strongly increased, with a particularly striking induction of *PHD3*. These results are in line with previous publications (Appelhoff et al., 2004; Marxsen et al., 2004). Importantly, the infection with wild-type or Δ*dotA C. burnetii* did not alter the *PHD2* and *PHD3* expression level. The infection with both *C. burnetii* strains, but not LPS, resulted in *PHD1* upregulation under normoxic conditions (**Figure 5**). The mRNA level of *VHL* was increased by hypoxia, which was augmented by LPS and by infection with wild-type or Δ*dotA C. burnetii*. While neither the oxygen level nor the infection state influenced *FIH* and *p300* expression, we observed that LPS resulted in reduced expression of *FIH* under normoxia and hypoxia and of *p300* under normoxia (**Figure 5**). In addition, *CBP* expression was reduced in hypoxic conditions and under normoxia when infected with Δ*dotA C. burnetii* (**Figure 5**). These data demonstrate that a *C. burnetii* infection influenced *HIF1α* (H) and *PHD1* (N) mRNA levels, regardless of the T4SS and in a different manner than LPS. Furthermore, under normoxia, the infection with Δ*dotA C. burnetii*, but not with the wild-type, reduces *CBP* expression.

### *C. burnetii* Infection Supports the Switch to Glycolysis in Macrophages

**Figure 6:**
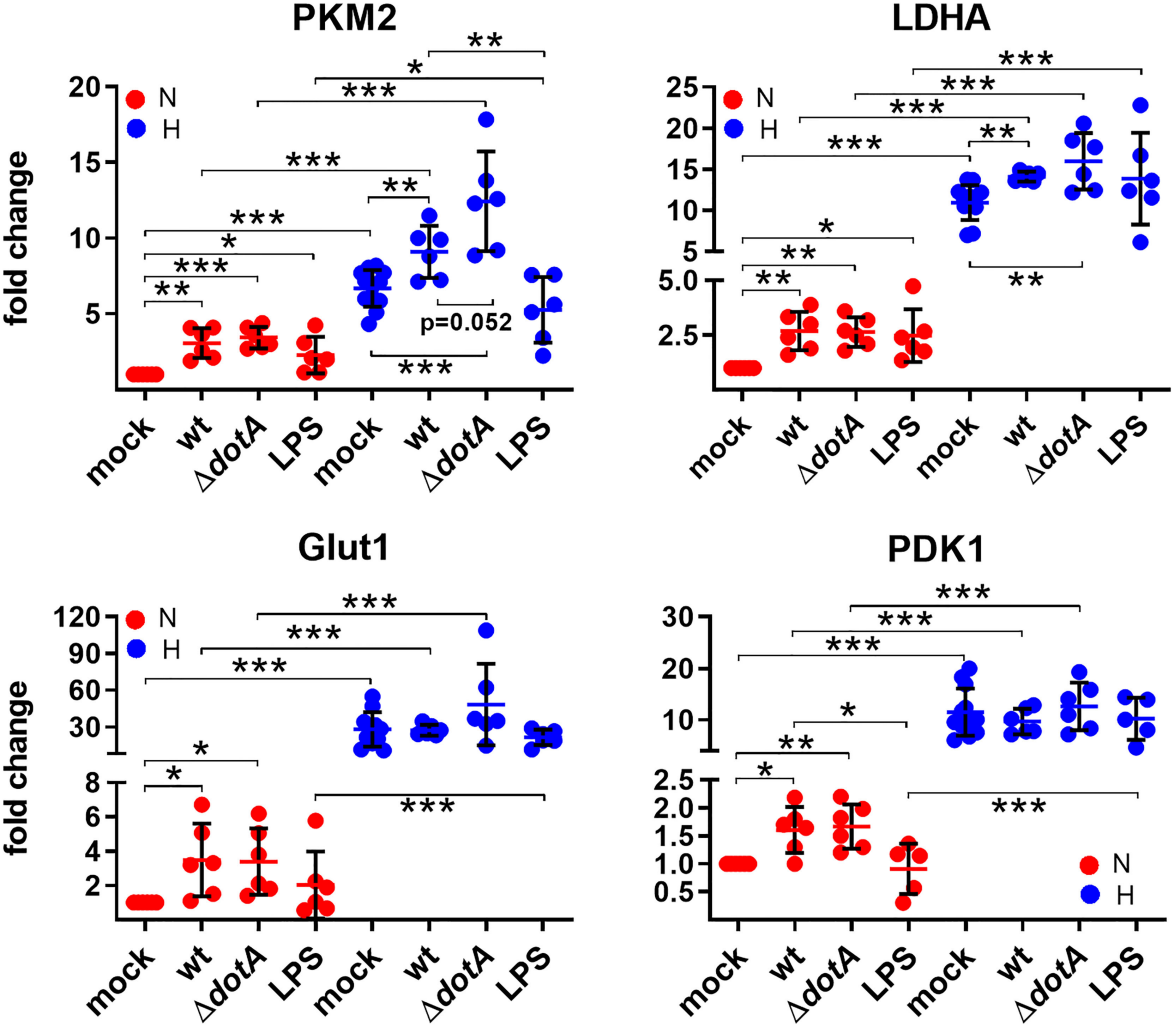
*C. burnetii*-infected macrophages reveal a shift to glycolysis. BMDM were either uninfected (mock), infected with *C. burnetii* (wt), the T4SS transposon mutant (Δ*dotA*), or treated with LPS (100 ng/ml) for 24 h under normoxia (N) and hypoxia (H). Using qRT-PCR, the gene expression of murine *PKM2*, *LDHA*, *Glut1*, and *PDK1* was analyzed. The data are shown as Mean ± SD of 2^-ΔΔCT values (using murine *HPRT* as a calibrator). Fold changes are shown relative to uninfected cells under N. The data shown for each of the *C. burnetii* (wt and Δ*dotA*) infection experiments and the LPS treatment experiment represent 3 independent experiments with biological duplicates. One sample t test or t test, n=5-6. ***p < 0.001, **p < 0.01, *p < 0.05.

HIF1α is an important transcription factor critical for cellular metabolism, for regulation of apoptosis and autophagy and for immune responses (Cramer et al., 2003; Corcoran and O'Neill, 2016; Knight and Stanley, 2019). Therefore, we analyzed the role of oxygen availability in combination with a *C. burnetii* infection or with LPS stimulation as a control on HIF1α target gene expression. First, we concentrated on metabolic genes. As shown in **Figure 6**, oxygen limitation resulted in upregulation of *PKM2*, *LDHA*, *Glut1* and *PDK1*. These factors are involved in glucose uptake (Glut1), generation of pyruvate (PKM2), conversion of pyruvate to lactate (LDHA), and inhibition of the conversion of pyruvate to acetyl-CoA (PDK1), which indirectly increases the conversion of pyruvate to lactate. These data are in line with previous findings showing that HIF1α is essential for the switch to glycolysis in macrophages (Cramer et al., 2003). The infection with wild-type and Δ*dotA C. burnetii* increases the expression of *PKM2* and *LDHA* in normoxic and hypoxic BMDM, and the expression of *Glut1* and *PDK1* only in normoxic BMDM (**Figure 6**). Importantly, treatment with LPS resulted in a similar modulation of the expression of the metabolic genes analyzed. There were only two exceptions: 1) the infection with *C. burnetii* induced a significantly higher expression of PKM2 under hypoxia than LPS; 2) the infection with *C. burnetii* induced a significantly higher expression of PDK1 under normoxia than LPS. These data suggest that an infection partially promotes upregulation of the metabolic target genes analyzed. How *C. burnetii* supports the switch to glycolysis mechanistically is unknown. LPS might play a role in this shift (**Figure 6**), which is in line with previous reports (Rodriguez-Prados et al., 2010).

### *C. burnetii* Infection and Hypoxia Independently Result in a More Pro-Apoptotic Signature

**Figure 7:**
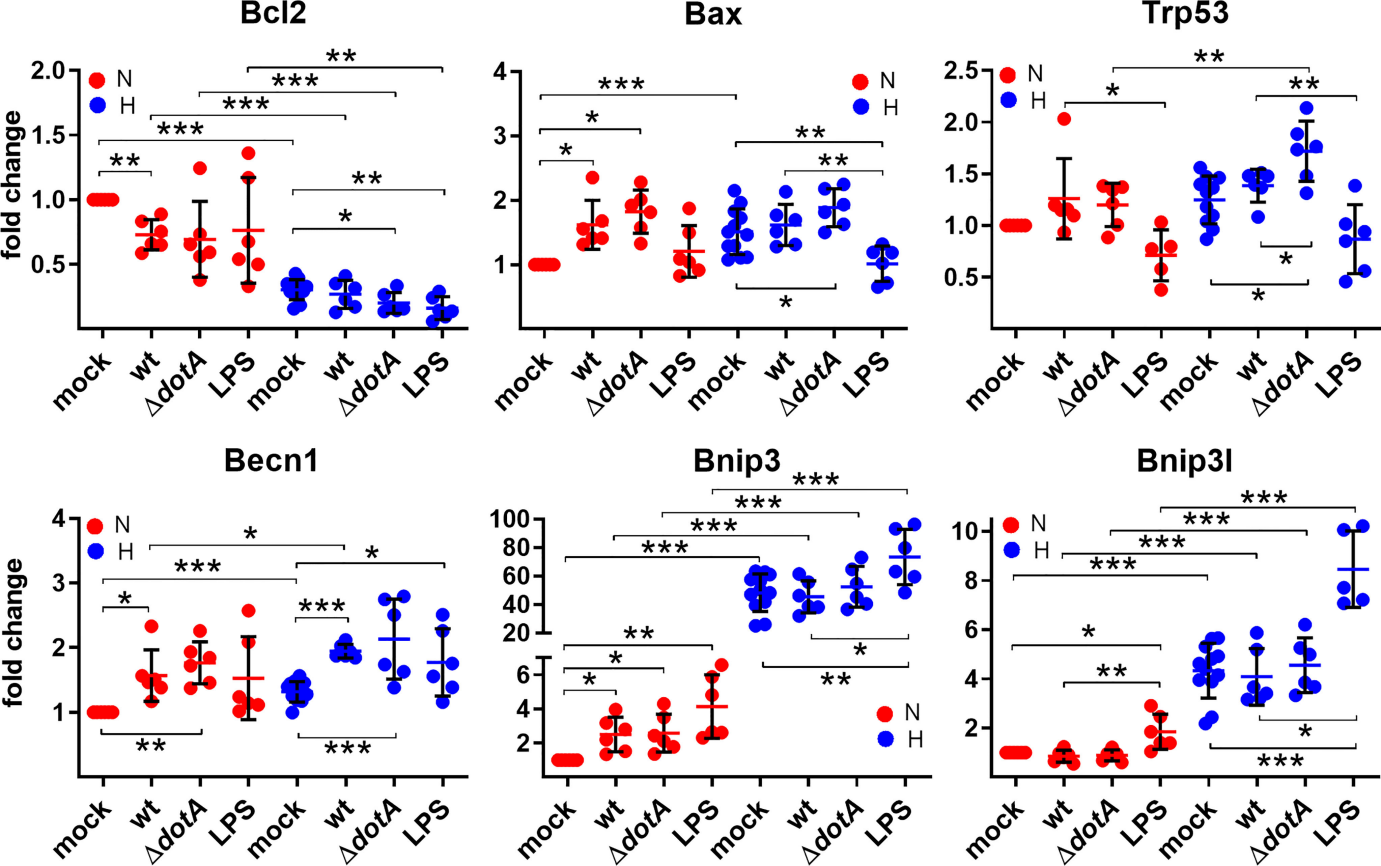
A pro-apoptotic expression signature is more prominent in *C. burnetii*-infected or hypoxic macrophages. BMDM were either uninfected (mock), infected with *C. burnetii* (wt), the T4SS transposon mutant (Δ*dotA*), or treated with LPS (100 ng/ml) for 24 h under normoxia (N) and hypoxia (H). Using qRT-PCR, the gene expression of murine *Bcl2*, *Bax*, *Trp53*, *Becn1*, *Bnip3*, and *Bnip3l* was analyzed. The data are depicted as Mean ± SD of 2^-ΔΔCT values (using murine *HPRT* as a calibrator). Fold changes are shown relative to uninfected cells under N. The data shown for each of the *C. burnetii* (wt and Δ*dotA*) infection experiments and the LPS treatment experiment represent 3 independent experiments with biological duplicates. One sample t test or t test, n=5-6. ***p < 0.001, **p < 0.01, *p < 0.05.

Next, we analyzed HIF1α target genes involved in regulating apoptotic and autophagic cell death. We then analyzed the mRNA levels of anti-apoptotic Bcl-2, pro-apoptotic Bax and p53, which regulates ~500 target genes, thereby influencing DNA repair, cell cycle arrest, metabolism and cell death (Aubrey et al., 2018). While hypoxia decreased the expression of anti-apoptotic *Bcl-2*, it increased the expression of *Bax*. The infection with wild-type *C. burnetii* did not alter the transcription levels of *Bcl-2* and *Bax* under hypoxia. However, infection with Δ*dotA C. burnetii* resulted in down-regulation of Bcl-2 and upregulation of Bax under hypoxia. Under normoxia, the infection resulted in down-regulation of *Bcl-2* and up-regulation of *Bax* regardless of the genotype of the pathogen (**Figure 7**). This result was unexpected, as *C. burnetii* displays anti-apoptotic activity, and no influence on Bcl-2 and Bax protein levels was determined (Lührmann and Roy, 2007; Voth et al., 2007; Cordsmeier et al., 2019). However, this might be due to different cell types, primary versus cell lines, used.

Hypoxia and HIF1α regulate p53 in several ways and *vice versa* (Zhang et al., 2021). We did not find an influence of hypoxia on *p53* transcription level, but the infection under hypoxia resulted in an increased *p53* expression (**Figure 7**). Importantly, cells infected with the Δ*dotA* mutant showed a significant higher expression of *p53* compared to cells infected with wild-type *C. burnetii* (**Figure 7**).

This might be due to an increased HIF1α level in cells infected with the Δ*dotA* mutant (**Figures 4A, B**), but independent of LPS signaling, as LPS resulted in downregulation of *p53* expression under normoxia and hypoxia.

While analyzing genes involved in autophagic cell death induction, we observed an upregulation of *Beclin 1*, *Bnip3* and *Bnip3l* by hypoxia. The infection influenced the expression level of *Beclin 1*, both under normoxia and hypoxia, similarly as did LPS.

*Bnip3* expression was only upregulated by a *C. burnetii* infection under normoxia, but not under hypoxia, while *Bnip3l* expression was not modulated by the infection at all. Importantly, LPS treatment resulted in significant upregulation of *Bnip3* and *Bnip3l* under hypoxia, demonstrating that *C. burnetii*-induced expression modulation of the genes analyzed was partially independent of LPS signaling (**Figure 7**). These data suggest that hypoxia and the infection with *C. burnetii* affect the apoptosis-regulators analyzed independently towards a more pro-apoptotic signature.

### *C. burnetii* Infection Induces an Upregulation of Inflammatory Genes, Which is Shifted Under Hypoxia Towards a Pro-Inflammatory Signature

**Figure 8:**
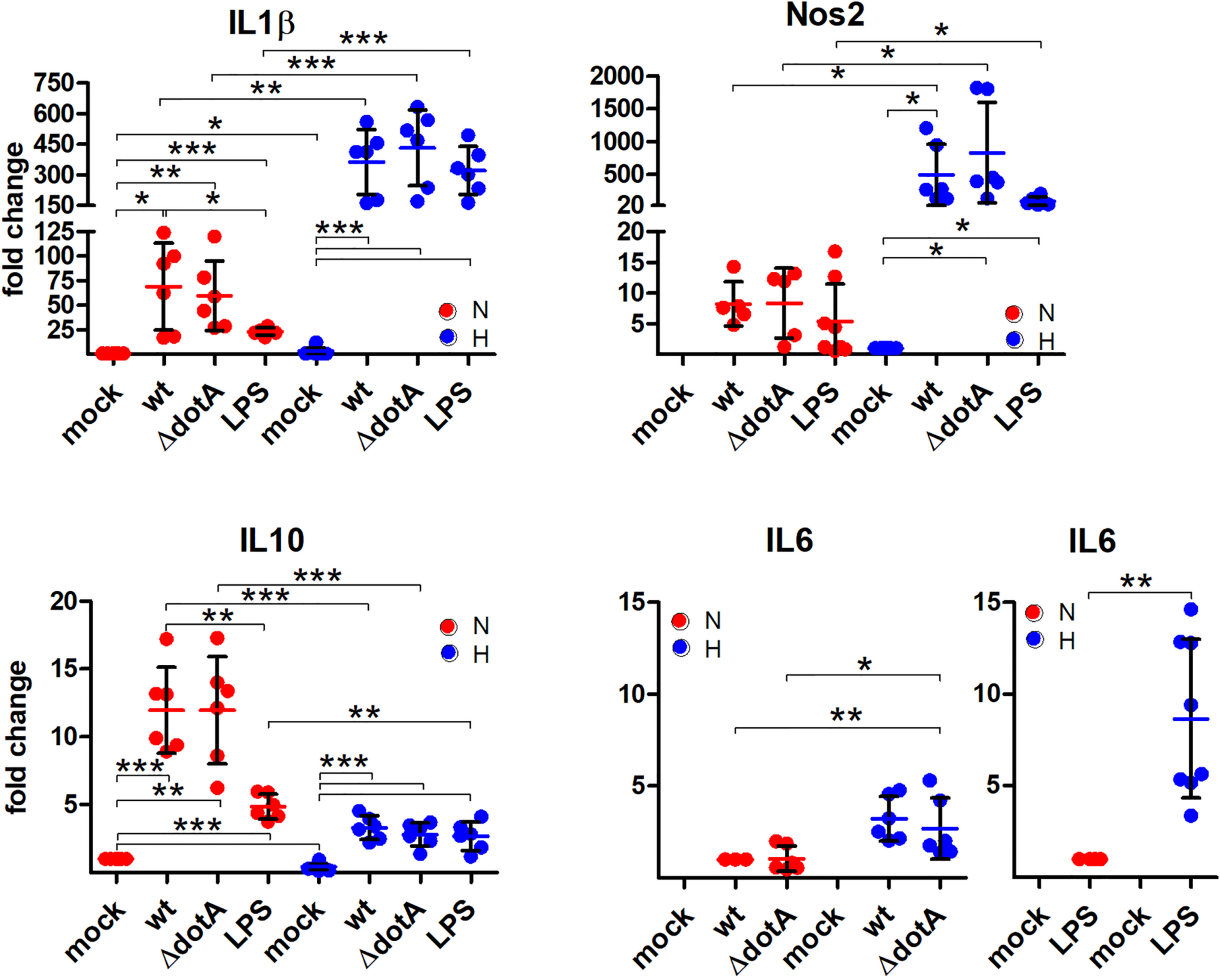
A pro-inflammatory profile is observed through *C. burnetii* infection under hypoxia. BMDM were either uninfected (mock), infected with *C. burnetii* (wt), the T4SS transposon mutant (Δ*dotA*), or treated with LPS (100 ng/ml) for 24 h under normoxia (N) and hypoxia (H). Using qRT-PCR, the gene expression of murine *IL1β*, *Nos2*, *IL10*, and *IL6* was analyzed. The data are plotted as Mean ± SD of 2^-ΔΔCT values (using murine *HPRT* as a calibrator). In case of *IL1β* and *IL10*, fold changes are shown relative to uninfected cells under N, while for *Nos2* they are represented relative to uninfected cells under H. For *IL6*, fold changes are shown relative to wt-infected cells or LPS-treated cells under N. The data shown for each of the *C. burnetii* (wt and Δ*dotA*) infection experiments and the LPS treatment experiment represent 3 independent experiments with biological duplicates. One sample t test or t test, n=5-6. ***p < 0.001, **p < 0.01, *p < 0.05.

Next, we analyzed the role of hypoxia and/or a *C. burnetii* infection on the transcription of inflammatory genes. We analyzed the pro-inflammatory HIF1α target genes *IL1β*, *IL6*, *Nos2* and the anti-inflammatory gene *IL10*. In the absence of infection, we detected an increase of *IL1β* and a decrease in *IL10*, when comparing normoxia versus hypoxia (**Figure 8**), which is in line with the observation that the HIF pathway regulates cytokine production in multiple cell types (Malkov et al., 2021). In contrast, the infection increased the expression of all genes analyzed. While the expression of the pro-inflammatory genes was increased under hypoxia compared to under normoxia, this was the opposite for the anti-inflammatory gene *IL10* (**Figure 8**). Importantly, the *C. burnetii* infection resulted in significantly stronger induction of *IL10* under normoxia than the LPS treatment. In contrast, the combination of hypoxia and LPS treatment resulted in an upregulation of *IL6* expression by ~9 fold, while the combination of hypoxia and *C. burnetii* infection only led to a ~3 fold upregulation. However, we did not detect a difference between BMDM infected with the wild-type or the T4SS mutant, indicating that the effect of *C. burnetii* on inflammatory HIF1α-target genes is independent of the T4SS. Thus, our data indicates that the *C. burnetii* infection results in upregulation of pro- and anti-inflammatory genes. Importantly, under hypoxia, the expression profile of the genes analyzed shifts towards a pronounced pro-inflammatory signature.

## Discussion

While HIF1α was first identified as an essential regulator of hypoxia (Majmundar et al., 2010), it is now clear that this transcription factor is also activated by several human pathogens even under normoxia (Werth et al., 2010). As HIF1α regulates cellular metabolism, immune cell activity, and inflammatory responses (Knight and Stanley, 2019), it is a central player during host-pathogen interaction.

Thus, it is not surprising that several pathogens have evolved proteins that modulate HIF1α activity (Knight and Stanley, 2019). For example, the *Salmonella enterica* siderophore Sal activates HIF1 (Hartmann et al., 2008), as does BadA from *Bartonella henselae* (Riess et al., 2004). In contrast, the AQ signaling molecule from *Pseudomonas aeruginosa* leads to proteasomal degradation of HIF1 (Legendre et al., 2012). These examples demonstrate that dependent on the nature and requirements of the respective pathogen, the ability to interfere with HIF1 is distinct.

We recently showed that HIF1α is responsible for controlling *C. burnetii* infection in an *in vitro* infection model using primary murine and human macrophages (Hayek et al., 2019). However, although HIF1α was proven beneficial for limiting bacterial replication (Hayek et al., 2021), it did not affect the cell´s ability to clear *C. burnetii* (Hayek et al., 2019). This is in line with previous observations that hypoxia and/or HIF1α induce a state of bacterial persistence and dormancy, which might impair bacterial clearance and allow the emergence of reoccurring or chronic infections (Sershen et al., 2016; Hayek et al., 2019; Hayek et al., 2021).

Here, we show that *C. burnetii* is able to curtail HIF1α, which depends on bacterial viability and protein synthesis (**Figures 3A, B**). The data suggests that the T4SS is involved (**Figures 4A, B**) indicating that a bacterial factor is required for this activity. The T4SS, an essential virulence factor of *C. burnetii*, injects over 150 effector proteins into the host cell to manipulate several host cell pathways enabling the pathogen to survive and replicate intracellularly (Lührmann et al., 2017). Only a few of these effector proteins have been functionally characterized. They interfere with host cell transcription, apoptosis, pyroptosis, ER stress, autophagy, and vesicular trafficking (Cordsmeier et al., 2019; Burette and Bonazzi, 2020b; Thomas et al., 2020; Dragan and Voth, 2020). The effector protein(s) involved in destabilizing HIF1α is currently unknown. The reason why increasing infection rates, which most likely result in increased numbers of secreted effector proteins, did not result in increased HIF1α degradation (**Figures 2B, C**), is currently unknown. It might be the balance between activation by PAMPs and dampening by effector proteins. As we could not show the biological consequence of the T4SS-dependent HIF1α destabilization (**Figures 5** – **8**), we hypothesize that HIF1α destabilization might be a side effect and not the primary function of a so far unknown effector protein. Thus, an effector protein interfering with the NF-κB signaling pathway might be involved, as NF-κB regulates HIF1α (Rius et al., 2008). Importantly, NF-κB modulation by the *C. burnetii* T4SS has been described (Mahapatra et al., 2016) and recently the *C. burnetii* T4SS effector protein NopA was identified to perturb NF-κB activation (Burette et al., 2020a). Thus, it can be speculated that NopA or a so far unknown effector protein might be indirectly involved in HIF1α activation. The increased level of *HIF1α* in cells infected with the T4SS mutant (Δ*dotA*) in comparison to cells infected with wild-type *C. burnetii* did not correlate with differences in the expression levels of HIF1α modulators (**Figure 5**), suggesting that a so far unknown effector protein might not interfere with the expression of HIF1α modulators. It might be possible that the effector protein interferes with the enzymatic activity of the PHDs or the availability of PHD co-factors (Siegert et al., 2015). Further research will be necessary to determine the molecular mechanisms leading to T4SS-dependent reduction of *C. burnetii*-induced HIF1α stabilization.

Nevertheless, we did not detect a difference in the expression of most of the HIF1α target genes analyzed in BMDM infected with either wild-type *C. burnetii* or the Δ*dotA* mutant (**Figures 6**– **8**), suggesting that the HIF1α protein level does not correlate with the level of HIF1α target gene expression. This was an unexpected finding, as correlation between HIF1α protein level and HIF1α target gene expression has been reported (Lee and Thorgeirsson, 2004; Lv et al., 2021). However, those reports analyzed the role of HIF1α in cancer or in cell lines, while we analyzed the role of HIF1α in primary cells during infection. Infected tissue is commonly found to be hypoxic, which triggers HIF1α stabilization (Jantsch and Schödel, 2015), and pathogens or their products are known to trigger HIF1α accumulation also under normoxia. In addition, bacterial products also activate transcription factors that might act synergistically or antagonistically with HIF1α (Hayek et al., 2021). Importantly, our data clearly demonstrates that the *C. burnetii* infection under hypoxia leads to upregulation of the pro-inflammatory genes *IL1β*, *IL6* and *Nos2*, and to downregulation of the anti-inflammatory gene *IL10* (**Figure 8**). This is in line with reports that HIF1α is required for mounting a pro-inflammatory response to bacterial and fungal pathogens (Peyssonnaux et al., 2005; Tannahill et al., 2013; Mills et al., 2016; Li et al., 2018). Especially the increased expression of *Nos2* and *IL1β* might be of biological consequence for the *C. burnetii* infection. The homodimeric enzyme NOS2 converts L-arginine and oxygen into L-citrulline and nitric oxide (NO) (Bogdan, 2015). The latter is important for controlling bacterial infections (Nathan and Shiloh, 2000), including a *C. burnetii* infection (Howe et al., 2002; Zamboni and Rabinovitch, 2003; Brennan et al., 2004). IL1β is produced as an inactive pro-form, which has to be cleaved to its active form following inflammasome activation (Dinarello, 2018). *C. burnetii* avoids activation of the inflammasome, and thus, pyroptosis (Cunha et al., 2015; Delaney et al., 2021). However, whether *C. burnetii* is able to prevent IL1β secretion induced by potent inflammasome stimuli has to be clarified, as conflicting reports exist (Cunha et al., 2015; Delaney et al., 2021). Of note, NO was found to inhibit the NLRP3 inflammasome-dependent processing of IL1β (Mishra et al., 2013). Thus, it will be of importance to analyze whether not only the expression of IL1β is increased, but also its secretion. In addition, we have to elucidate whether the increased levels of NO in hypoxic *C. burnetii* infected BMDM might inhibit IL1β processing and secretion.

In summary, our data demonstrate that *C. burnetii* influences HIF1α stability and activity. As HIF1α is important for mounting anti-bacterial responses, this might have consequences for the host-pathogen interaction and, thus, disease outcome.

## Data Availability Statement

The original contributions presented in the study are included in the article. Further inquiries can be directed to the corresponding author.

## Author Contributions

IH and MS performed the experiments and analyzed the data. AL and IH conceived the study. AL obtained funding, supervised the study and drafted the manuscript. All authors contributed to the writing of the manuscript.

## Funding

This work was supported by the Deutsche Forschungsgemeinschaft (DFG) through the Collaborative Research Initiative 1181 (CRC1181) project A06 to AL. IH was partially funded by the Bavarian Equal Opportunities Sponsorship – Realisierung von Chancengleichheit von Frauen in Forschung und Lehre (FFL) – Realization Equal Opportunities for Women in Research and Teaching.

## Conflict of Interest

The authors declare that the research was conducted in the absence of any commercial or financial relationships that could be construed as a potential conflict of interest.

## Publisher’s Note

All claims expressed in this article are solely those of the authors and do not necessarily represent those of their affiliated organizations, or those of the publisher, the editors and the reviewers. Any product that may be evaluated in this article, or claim that may be made by its manufacturer, is not guaranteed or endorsed by the publisher.
